# SGLT2 inhibitors and finerenone in non-diabetic CKD: a step into the (near) future?

**DOI:** 10.1093/ckj/sfad272

**Published:** 2023-10-23

**Authors:** Marieta P Theodorakopoulou, Pantelis Sarafidis

**Affiliations:** First Department of Nephrology, Hippokration Hospital, Aristotle University of Thessaloniki, Thessaloniki, Greece; First Department of Nephrology, Hippokration Hospital, Aristotle University of Thessaloniki, Thessaloniki, Greece

Chronic kidney disease (CKD) is a major public health problem affecting 850 million people worldwide [1]. It is a major risk factor for increased cardiovascular and all-cause mortality and is anticipated to become the fifth global cause of death by the year 2040 [2]. Increased rates of CKD progression towards end-stage kidney disease (ESKD) requiring kidney replacement therapy is anticipated to place a huge burden on healthcare systems [3]. As such, delaying both the onset and progression of CKD should be a main goal for public health worldwide [4].

Lifestyle modifications, control of blood pressure (BP), and glycemic and lipid abnormalities, along with the use of an angiotensin-converting enzyme inhibitor (ACEi) or an angiotensin-receptor blocker (ARB) have been the cornerstone of CKD treatment for decades [5, 6]. In recent years, several studies with hard kidney outcomes have shown important nephro- and cardioprotection with sodium–glucose cotransporter 2 inhibitors (SGLT2i) in patients with CKD with or without diabetes. In the “Canagliflozin and Renal Events in Diabetes with Established Nephropathy Clinical Evaluation” (CREDENCE) study in proteinuric CKD with type 2 diabetes, canagliflozin was associated with 30% lower risk for the primary renal outcome [7]; the “Dapagliflozin and Prevention of Adverse Outcomes in Chronic Kidney Disease” (DAPA-CKD) trial confirmed and expanded the above by showing that dapagliflozin reduces the risk of a composite of sustained decline in estimated glomerular filtration rate (eGFR) ≥50%, ESKD, or death from renal or cardiovascular causes, in patients with diabetic or non-diabetic CKD with a urine albumin-to-creatinine ratio (UACR) ≥200 mg/g by 44%. Most recently, in the “Study of Heart and Kidney Protection with Empagliflozin” (EMPA-KIDNEY) trial, empagliflozin reduced the composite primary endpoint of CKD progression (ESKD, sustained decrease in eGFR to <10 mL/min/1.73 m^2^ or eGFR of ≥40% from baseline, or death from renal causes) or cardiovascular death by 28% in a diverse population of 6609 CKD patients with normo-, moderately increased and severely increased albuminuria and eGFR levels between 20 and 90 mL/min/1.73 m^2^, of whom >50% were not diabetic [8]. Of note, after the initial eGFR dip, data on chronic eGFR slopes were consistent with benefit at any eGFR or UACR level, a fact translating in delaying kidney replacement therapy by 2–27 years compared with standard therapy alone, depending on baseline risk [9].

In parallel with the above, within the past few years, the “Efficacy and Safety of Finerenone in Subjects With Type 2 Diabetes Mellitus and Diabetic Kidney Disease” (FIDELIO-DKD) and “Efficacy and Safety of Finerenone in Subjects With Type 2 Diabetes Mellitus and the Clinical Diagnosis of Diabetic Kidney Disease” (FIGARO-DKD) trials demonstrated that addition of finerenone, a novel, nonsteroidal, selective mineralocorticoid receptor antagonist on top of optimal medical treatment led to reduction of renal and cardiovascular outcomes compared with placebo in patients with CKD, moderately increased and severely increased albuminuria and type 2 diabetes [10, 11]. In both trials an early dip in eGFR followed by a lower rate of eGFR loss and an early reduction in UACR by 40% which persisted over time were noted in the finerenone arms [10, 11], even in distinct patient subgroups, such as those with CKD stage 4 [12].

Previous evidence has suggested that the combination of SGLT2i and mineralocorticoid receptor antagonists may result in more pronounced kidney benefits. In a preclinical rat model of hypertension‐induced cardiorenal disease, co‐administration of finerenone with empagliflozin led to efficacious reduction in several functional parameters such as proteinuria and BP levels, cardiac and renal lesions as determined by histopathology, and mortality, compared with monotherapy with each drug alone [13]. Furthermore, in a previous open-label randomized crossover trial in patients with pre-dialysis CKD, combining dapagliflozin with eplerenone resulted in a robust additive albuminuric-lowering effect, whereas the changes in albuminuria did not correlate between the two different arms [14]. The FIDELITY analysis was a pre-specified pooled analysis of both FIDELIO-DKD and FIGARO-DKD trials and included 13 026 patients, with mean eGFR 57.6 mL/min/1.73 m^2^ and median UACR 515 mg/g. In a hypothesis-generated sub-analysis examining the influence of concomitant treatment with an SGLT2i at baseline or at any time concomitant with study treatment on cardiorenal outcomes [15], the hazard ratios (HRs) with finerenone versus placebo for the kidney composite outcome were 0.80 [95% confidence interval (CI) 0.69–0.92] in patients not receiving and 0.42 (95% CI 0.16–1.08) in patients receiving an SGLT2i at baseline. For the cardiovascular composite, the HRs were 0.87 (95% CI 0.79–0.96) without and 0.67 (95% CI 0.42–1.07) with SGLT2i at baseline. Patients receiving an SGLT2i at baseline had lower incidence of hyperkalemia in both the placebo and finerenone groups [16]. Of note, the differences in chronic eGFR slope from Month 4 to the end of study for patients using an SGLT2i at baseline were –1.92 versus –3.45 mL/min/1.73 m^2^ for finerenone and placebo, whereas for those without SGLT2i were –2.54 versus –3.52 mL/min/1.73 m^2^, respectively (Fig. 1) [15]. Several hypotheses for these synergistic effects of the two classes on cardiorenal outcomes were proposed. Apart from shared mechanism that may be associated with delayed CKD progression (i.e. the hemodynamic actions mediating reduction in intraglomerular pressure), there could be also blockade of distinct pathways leading to renal inflammation and fibrosis [16, 17]. In addition, a protective effect of the SGLT2i against hyperkalemia could be present, enabling patients to stay longer on finerenone treatment [16, 17].

**Figure 1:**
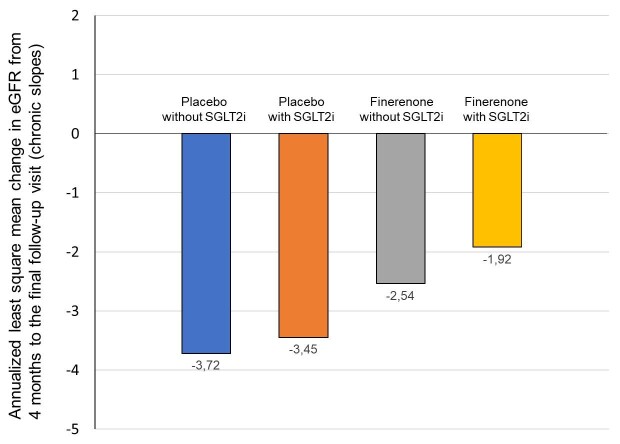
Annualized least square mean change in eGFR from 4 months to final visit in the FIDELITY pooled analysis, stratified by the concomitant use of an SGLTi at baseline (modified from Rossing *et al*. [15]).

In this issue of *Clinical Kidney Journal*, Mårup *et al*. report on a pilot single-center, open-label, randomized clinical trial that investigated the short-term effects of finerenone and dapagliflozin separately and in combination on albuminuria, eGFR levels and safety parameters in patients with non-diabetic, proteinuric CKD [18]. It included 20 patients with CKD with eGFR 25–45 mL/min/1.73 m^2^ and UACR 150–2000 mg/g on maximal tolerated dose of ACEi/ARB. Participants received either finerenone 20 mg/day or dapagliflozin 10 mg/day for 4 weeks, followed by combination therapy for another 4 weeks. Data were collected at baseline, and at 4 and 8 weeks. The primary endpoint was the change in UACR from randomization to 8 weeks. Secondary endpoints were the change in UACR, albuminuria measured on a 24-h urine sample, office BP, eGFR and measured GFR (technetium-99m-DTPA clearance) during each 4-week treatment period. The results demonstrated that finerenone alone or in addition to dapagliflozin resulted in –24% (*P *= .004 versus baseline) and –34% (*P *= .002 versus baseline) reduction in UACR, respectively, but the difference between 4 and 8 weeks was not significant (*P *= .35). Dapagliflozin alone or in addition to finerenone resulted in –8% (*P *= .34 versus baseline) and –10% change (*P *= .35 versus baseline) in UACR, at 4 and 8 weeks respectively, again without significant difference between the two time-points (*P *= .85). The change in UACR from baseline to 8 weeks in both groups combined was –36% (95% CI –46% to –24%). For the whole study population, the estimated combined effect on UACR of finerenone and dapagliflozin at 4 weeks, based on multiplying the independent effects of each drug alone (baseline to Week 4 in both groups, respectively) was a change of –30%, whereas the observed combined mean effect was –36%, with no significant difference between the calculated and the observed effects of combination therapy (*P *= .47). Furthermore, reductions in albuminuria observed in individual patients with either finerenone or dapagliflozin were only weakly correlated in both groups, and thus, the effect of the initial administered drug on UACR did not predict the effect on UACR of the subsequent drug. At 8 weeks, systolic BP and GFR were reduced by 10 mmHg (95% CI 6–13 mmHg) and 7 mL/min (95% CI 5–8 mL/min). Adverse effects were minimal and neither drug required discontinuation in any participant during the study period.

This pilot study is the first randomized controlled trial indicating a beneficial effect of each of finerenone and dapagliflozin, as well as their combination, on albuminuria in patients with non-diabetic CKD. The effects of either drug were independent of previous treatment with the other, and the effect of the combination was at least equal to the calculated, combined effects of the independent drugs. However, the study had several limitations. The small sample size and the short study duration did not enable evaluation of possible differences on albuminuria between these two agents during the first phase of treatment, nor evaluation of differences between the combined phase of treatment and each individual drugs. Furthermore, benefits on albuminuria with any relevant agent during an 8-week treatment are probably related to the hemodynamically mediated eGFR “dip” within the first weeks of drug initiation, and not to long-term effects relevant to structural kidney changes. Most importantly, the study could not and obviously did not evaluate any effects on more robust surrogate markers (i.e. eGFR slopes) and hard outcomes, such as progression to ESKD.

Some of these questions are expected to be answered in the near future. The “COmbinatioN effect of FInerenone anD EmpaglifloziN in participants with CKD and type 2 diabetes using a UACR Endpoint” (CONFIDENCE) study [19], is an ongoing randomized, double-blind, multicenter, parallel-group, phase 2 study enrolling 807 adults with type 2 diabetes, stage 2–3 CKD and UACR 300–5000 mg/g that also uses the change in UACR as the primary outcome and as an indicator for progression of CKD. The total study duration is approximately 9 months and the first results are expected within 2024. With regards to patients with non-diabetic CKD, some evidence in this field is expected after the completion of the “Finerenone In Non-Diabetic Chronic Kidney Disease” (FIND-CKD) study (ClinicalTrials.gov: NCT05047263), a randomized, double-blind, placebo-controlled, parallel-group trial randomizing patients with non-diabetic CKD with eGFR 25–90 mL/min/1.73 m^2^ and UACR 200–3500 mg/g on stable and maximum tolerated dose of an ACEi/ARB to finerenone 10 or 20 mg or placebo with a primary endpoint of the change total slope of eGFR from baseline to 32 months. The study was started in September 2021 and is expected to be completed in November 2025. Secondary analyses according to concomitant use of an SGLT2i, expected to be around 15%–20% at baseline, are awaited to shed more light in the field.

In conclusion, the study from Mårup *et al*. is an interesting addition in the literature suggesting that finerenone, dapagliflozin and their combination can reduce albuminuria on top of RAS blockade over a short-term period in patients with non-diabetic CKD. Whether the effect of combination of these classes is more potent than the use of each individual class, and whether this translates in further meaningful reductions in harder kidney outcomes in patients with CKD remains to be examined in future trials.

## References

[1] Jager KJ , Kovesdy C, Langham R et al. A single number for advocacy and communication-worldwide more than 850 million individuals have kidney diseases. Nephrol Dial Transplant 2019;34:1803–5. 10.1093/ndt/gfz174 31566230

[2] Foreman KJ , Marquez N, Dolgert A et al. Forecasting life expectancy, years of life lost, and all-cause and cause-specific mortality for 250 causes of death: reference and alternative scenarios for 2016-40 for 195 countries and territories. Lancet 2018;392:2052–90. 10.1016/S0140-6736(18)31694-5 30340847 PMC6227505

[3] Klarenbach SW , Tonelli M, Chui B et al. Economic evaluation of dialysis therapies. Nat Rev Nephrol 2014;10:644–52. 10.1038/nrneph.2014.145 25157840

[4] Ruiz-Ortega M , Rayego-Mateos S, Lamas S et al. Targeting the progression of chronic kidney disease. Nat Rev Nephrol 2020;16:269–88. 10.1038/s41581-019-0248-y 32060481

[5] Sarafidis PA , Ruilope LM. Aggressive blood pressure reduction and renin-angiotensin system blockade in chronic kidney disease: time for re-evaluation? Kidney Int 2014;85:536–46. 10.1038/ki.2013.355 24048382

[6] Mancia G , Kreutz R, Brunström M et al. 2023 ESH Guidelines for the management of arterial hypertension The Task Force for the management of arterial hypertension of the European Society of Hypertension Endorsed by the International Society of Hypertension (ISH) and the European Renal Association (ERA). J Hypertens 2023. 10.1097/HJH.0000000000003480 (epub ahead of print). 37345492

[7] Perkovic V , Jardine MJ, Neal B et al. Canagliflozin and renal outcomes in type 2 diabetes and nephropathy. N Engl J Med 2019;380:2295–306. 10.1056/NEJMoa1811744 30990260

[8] The EMPA-KIDNEY Collaborative Group; Herrington WG, Staplin N, Wanner C et al. Empagliflozin in patients with chronic kidney disease. N Engl J Med 2023;388:117–27. 36331190 10.1056/NEJMoa2204233 PMC7614055

[9] Fernández-Fernandez B , Sarafidis P, Soler MJ et al. EMPA-KIDNEY: expanding the range of kidney protection by SGLT2 inhibitors. Clin Kidney J 2023;16:1187–98. 10.1093/ckj/sfad082 37529652 PMC10387399

[10] Bakris GL , Agarwal R, Anker SD et al. Effect of Finerenone on chronic kidney disease outcomes in type 2 diabetes. N Engl J Med 2020;383:2219–29. 10.1056/NEJMoa2025845 33264825

[11] Pitt B , Filippatos G, Agarwal R et al. Cardiovascular events with finerenone in kidney disease and type 2 diabetes. N Engl J Med 2021;385:2252–63. 10.1056/NEJMoa2110956 34449181

[12] Sarafidis P , Agarwal R, Pitt B et al. Outcomes with finerenone in participants with stage 4 CKD and type 2 diabetes: a FIDELITY subgroup analysis. Clin J Am Soc Nephrol 2023;18:602–12. 10.2215/CJN.0000000000000149 36927680 PMC10278789

[13] Kolkhof P , Hartmann E, Freyberger A et al. Effects of finerenone combined with empagliflozin in a model of hypertension-induced end-organ damage. Am J Nephrol 2021;52:642–52. 10.1159/000516213 34111864 PMC8619789

[14] Provenzano M , Puchades MJ, Garofalo C et al. Albuminuria-lowering effect of dapagliflozin, eplerenone, and their combination in patients with chronic kidney disease: a randomized crossover clinical trial. J Am Soc Nephrol 2022;33:1569–80. 10.1681/ASN.2022020207 35440501 PMC9342643

[15] Rossing P , Anker SD, Filippatos G et al. Finerenone in patients with chronic kidney disease and type 2 diabetes by sodium-glucose cotransporter 2 inhibitor treatment: the FIDELITY analysis. Diabetes Care 2022;45:2991–8. 10.2337/dc22-0294 35972218 PMC9862372

[16] Sarafidis P , Iatridi F, Ferro C et al. Mineralocorticoid receptor antagonist use in chronic kidney disease with type 2 diabetes: a clinical practice document by the European Renal Best Practice (ERBP) board of the European Renal Association (ERA). Clin Kidney J 2023;sfad139. 10.1093/ckj/sfad139 (epub ahead of print). PMC10616462 37915899

[17] Mark PB , Sarafidis P, Ekart R et al. SGLT2i for evidence based cardiorenal protection in diabetic and non-diabetic chronic kidney disease: a comprehensive review by EURECA-m and ERBP working groups of ERA. Nephrol Dial Transplant 2023;gfad112. 10.1093/ndt/gfad112 (epub ahead of print). PMC10615631 37230946

[18] Mårup FH , Thomsen MB, Birn H. Additive effects of dapagliflozin and finerenone on albuminuria in non-diabetic CKD: an open-label randomized clinical trial. Clin Kidney J 2023;sfad249. (epub ahead of print) 10.1093/ckj/sfad249 38186886 PMC10768792

[19] Green JB , Mottl AK, Bakris G et al. Design of the COmbinatioN effect of FInerenone anD EmpaglifloziN in participants with chronic kidney disease and type 2 diabetes using a UACR Endpoint study (CONFIDENCE). Nephrol Dial Transplant 2023;38:894–903. 10.1093/ndt/gfac198 35700142 PMC10064838

